# Geopharmacosurveillance of reporting rates of events supposedly attributable to vaccination or immunization against COVID-19*

**DOI:** 10.1590/1518-8345.7509.4539

**Published:** 2025-05-02

**Authors:** Thays Cristina Pereira Barbosa, Gabriela Lourença Martins do Nascimento, Luiz Henrique Arroyo, Ricardo Alexandre Arcêncio, Valéria Conceição de Oliveira, Eliete Albano de Azevedo Guimarães

**Affiliations:** 1 Universidade Federal de São João del-Rei, Campus Centro-Oeste Dona Lindu, Divinópolis, MG, Brazil; 2 Scholarship holder at the Conselho Nacional de Desenvolvimento Científico e Tecnológico (CNPq), Brazil; 3 Ministério da Saúde, Secretaria de Vigilância em Saúde, Brasília, DF, Brazil; 4 Universidade de São Paulo, Escola de Enfermagem de Ribeirão Preto, PAHO/WHO Collaborating Centre for Nursing Research Development, Ribeirão Preto, SP, Brazil

**Keywords:** Vaccines, Adverse Event, COVID-19, Public Health Surveillance, Spatial Analysis, Ecological Studies

## Abstract

to analyze the spatial distribution of the reporting rate of events supposedly attributable to vaccination or immunization against COVID-19 and factors associated with achieving the target recommended by the Global Vaccine Action Plan.

ecological study that considered notifications from 853 municipalities in the state of Minas Gerais. A total of 34,027 notifications recorded in the *e-SUS Notifica* system were analyzed. Getis-Ord Gi* and Geographically weighted regression were performed to identify spatial clusters, compliance with at least 10 notifications and factors correlated with spatial distribution.

a heterogeneous distribution of reporting rates was observed throughout the state. A total of 20.3% of municipalities did not meet the recommended reporting target. The municipalities in the Northwest, Jequitinhonha and Vale do Aço macro-regions had the highest reporting rates in the state, while those in the East, East South and West macro-regions had the lowest rates. The number of nursing professionals per inhabitant (regression coefficient= 0.644; p< 0.01) and the percentage of families living in rural areas (regression coefficient= -0.013; p< 0.01) were associated with reporting rates.

the presence of clusters of low reporting rates highlights the need to implement integrated strategies adapted to the particularities of each region to enhance event reporting surveillance.

## Highlights

**(1)** Spatial heterogeneities of reporting rates in the state of Minas Gerais. **(2)** Identification of areas that require priority surveillance interventions. **(3)** Need to implement local actions to reduce underreporting of ESAVI. **(4)** Identification of factors associated with the ESAVI reporting rate. **(5)** Contribution to health policies aligned with global goals.

## Introduction

The World Health Organization (WHO) recognizes immunization as one of the main goals of the 2030 agenda, included in the third objective as a way to ensure a healthy life and promote well-being for all, at all ages^(1)^. Vaccination provides protection against a diverse range of infectious diseases, such as COVID-19. Although it is referred to as one of the safest and most effective preventive health interventions, vaccination is not exempt from Events Supposedly Attributable to Vaccination or Immunization (ESAVI)^(2-3)^.

An ESAVI is any abnormal laboratory symptom or disease, unfavorable and undesirable, whose occurrence occurs after vaccination, and which does not have a precise causal relationship with the use of a vaccine and the immunization process, and can be considered a serious adverse event (SAE) or a non-serious adverse event (NSAE)^(3)^.

Continuous surveillance of ESAVI is necessary to identify and mitigate risks related to vaccines and vaccination practices^(4)^. To this end, event reporting plays a crucial role, being an essential practice in surveillance^(5)^. This notification allows for detailed investigation of cases, facilitating understanding of the occurrence, causality and evolution of events. In addition, it enables the identification of risks, more vulnerable population groups and the contextual realities in which they are inserted, considering institutional/organizational, environmental, political and socioeconomic factors^(6-11)^.

The period analyzed in this study, from January to December 2021, was marked by the largest vaccination campaign in Brazil’s history, promoted by the Ministry of Health. Throughout that year, over 350 million doses of the COVID-19 vaccine were distributed nationwide. This massive mobilization resulted in a significant increase in the volume of notifications, reflecting the high demand for the vaccine and the intensification of vaccination actions^(12)^. This context highlights the importance of reporting as a surveillance performance indicator, allowing the identification of patterns and gaps in the adverse event recording and monitoring system (typing errors, incomplete fields and cases of underreporting)^(8-11,13-14)^.

Given the significance of ESAVI surveillance, the Global Vaccine Action Plan (GVAP) established the reporting rate as a performance indicator to assess progress in passive surveillance of vaccine safety^(13-14)^. The reporting rate analyzes the number of ESAVI notifications in a given region, based on its population, and the standard is that the region must reach at least 10 notifications^(15-16)^. The results of this measure are useful for comparative analyses between countries and regions, and can support the definition of goals for the progressive improvement of ESAVI surveillance, adapted to the particularities of each location^(16)^.

A comparative study of trends in global ESAVI reporting rates between 2000 and 2015 observed positive fluctuations in rates, with the number of countries with ESAVI reporting rates above 10 increasing from 8 (4%) in 2000 to 81 (42%) in 2015^(15)^. Following the same line, a research identified global progress in the proportion of countries that reported ESAVI between 2010 and 2019, increasing from 41.2% to 56.2%^(17)^. Moreover, a study conducted in the United States analyzed the reporting rate based on the population, revealing an increasing trend in ESAVI notifications in the country^(16)^.

In Brazil, no studies were identified that addressed the reporting rate to analyze the performance of immunization services in passive surveillance of ESAVI. Regarding notifications of ESAVI of COVID-19 vaccines in the country, these are carried out through *e-SUS Notifica*, a software platform that uses a reporting/investigation form, after spontaneous searching for the event identifier^(18-19)^. The monitoring of information on ESAVI allows for the identification of notifications, case follow-up, and improvement in the quality of information^(8-11)^.

However, underreporting of ESAVI has been a worrying challenge, highlighting the need to improve surveillance systems in Brazilian municipalities^(9,20)^. Underreporting can be attributed to several factors, such as insufficient health infrastructure, the conduct and lack of adequate training of professionals, lack of human resources, lack of supplies, lack of nursing supervision, work overload, lack of motivation, lack of awareness among the population, and geographic, cultural and economic aspects of the region^(21-26)^.

Given this scenario, it is essential to pay special attention and carry out strategic planning in line with the characteristics of each Brazilian location, in order to advance passive surveillance of vaccine safety.

This type of surveillance seeks to understand the interactions between medicines, the environment and human health, using spatial techniques to monitor and identify areas of potential risk and implement preventive and corrective measures targeted at specific regions. Geopharmacosurveillance of vaccines performs spatial monitoring of ESAVI or any other problem related to vaccination or immunization, helping to detect possible trends, clusters or areas with a higher incidence of the event^(27)^.

This study analyzed the spatial distribution of the reporting rate of events supposedly attributable to vaccination or immunization against COVID-19 and factors associated with achieving the target recommended by the Global Vaccine Action Plan.

## Method

### Study design

This is an ecological study, guided by the SQUIRE 2.0 tool (Standards for Quality Improvement Reporting Excellence).

### Study location

The study was carried out in Minas Gerais, the second most populous state in Brazil, with an estimated population of 21,411,923 million inhabitants, a degree of urbanization of 85.29%, a medium-high Human Development Index (HDI), and a diversified economy^(28)^. The choice of Minas Gerais to carry out this study is justified by its great demographic, social and economic diversity, in addition to being home to the largest number of municipalities in the country, which offers a rich and complex scenario for analysis. Furthermore, the partnership between the *Secretaria de Estado de Saúde* (SES-MG) and the *Universidade Federal de São João del-Rei* (UFSJ), through collaborative projects, ensures greater applicability and impact of the results.

**Figure 1 -:**
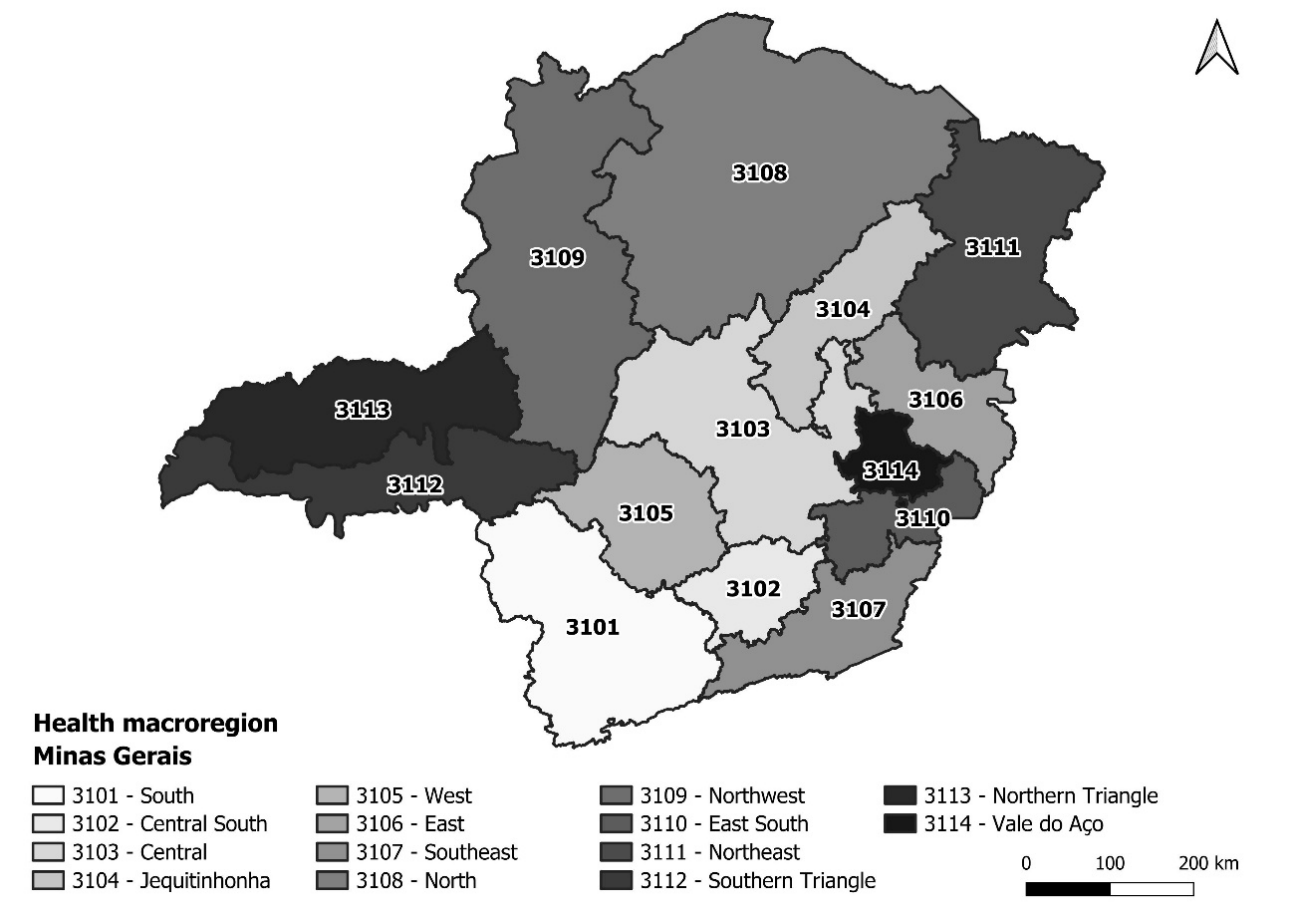
Health regions of the state of Minas Gerais, Brazil, 2022

The 853 municipalities are distributed across 14 health regions: South (3101), Central South (3102), Central (3103), *Jequitinhonha* (3104), West (3105), East (3106), Southeast (3107), North (3108), Northwest (3109), East South (3110), Northeast (3111), Southern Triangle (3112), Northern Triangle (3113) and *Vale do Aço* (3114) (Figure 1). These, in turn, encompass 89 health microregions and 28 Regional Health Superintendencies/Management Offices^(28-29)^.

For this study, the 853 municipalities in the 14 macro-regions of the state were established as territorial units of analysis (Figure 1).

### Population and data collection

The ESAVI notifications of COVID-19 vaccines, with and without closure, available on the electronic platform of the Department of Information Technology of the *Sistema Único de Saúde* (DATASUS, in Portuguese), *e-SUS Notifica* system^(19)^, between January and December 2021, were analyzed. The choice to focus on COVID-19 vaccination was driven by the relevance and urgency imposed by the pandemic, as well as its status as a recent event with a significant impact on public health. All 34,027 ESAVI notifications were included in this research, and immunization errors were excluded (n= 2663).

The outcome variable was the number of ESAVI notifications, with and without closure. The ESAVI notification is used to detect risks related to vaccines or vaccination practices, using the adverse event reporting/investigation form^(4)^. In this study, the ESAVI reporting rate was calculated, which consists of the number of ESAVI notifications divided by the population of a given region, multiplied by 100,000. The standard used to evaluate the reporting rate was a minimum number of 10 notifications per 100,000 inhabitants^(13-16)^.

**Figure 2 -:**
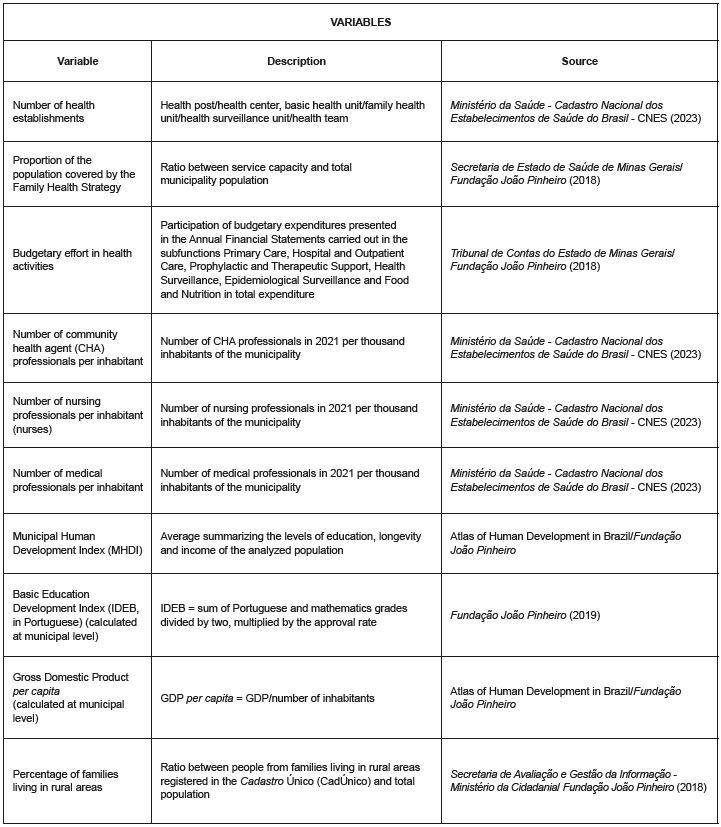
Description of exposure variables considered for the study. Minas Gerais, Brazil, 2023

In order to identify the factors associated with meeting ESAVI reporting rates, exposure variables were selected from the *Cadastro Nacional dos Estabelecimentos de Saúde do Brasil* (CNES), the database of the *Instituto Brasileiro de Geografia e Estatística* (IBGE), and the *Fundação João Pinheiro* (FJP), which were included in the logistic regression analyses (Figure 2).

### Data processing and analysis

Initially, in the exploratory phase, Microsoft Office Excel software, version 2016, was used to verify the consistency and quality of ESAVI reporting records.

In addition, the ESAVI reporting rate (number of ESAVI notifications divided by the population of a given region, multiplied by 100,000) was used to create maps for the Getis-Ord Gi* and Geographically weighted regression (GWR) analyses^(30-31)^.

The tool used to determine the distribution of ESAVI reporting rates in space was the GeoDa 1.20 software. This is a free software and code instrument designed to perform exploratory spatial data analysis (ESDA) methods, such as spatial autocorrelation statistics. It also provides a statistical module for Getis-Ord Gi* analysis. And the GWR analysis was performed by the free software R 4.3, for statistical computing and graphics^(32-34)^. For all spatial analyses, a first-order queen-type neighborhood matrix was used. All municipalities that share direct borders in any direction were considered.

Getis-Ord Gi* analysis was used to identify clusters of ESAVI reporting rates resulting from COVID-19 vaccines. The statistic consists of the ratio of the weighted average of the values of neighboring locations to the sum of all values, including the value of the specific location that is analyzed in an analytical window. Thus, values above the average are classified as areas of high occurrence, while values below the average suggest low occurrence. This analysis results in two classifications: one for municipalities with high, statistically significant values (high occurrence area/hot area - Hotspot) and another for municipalities with low values, also statistically significant (low occurrence area/cold area - Coldspot)^(31,35)^.

To identify the factors associated with the ESAVI reporting rate, a geographically weighted regression was conducted, considering explanatory variables that characterize the analyzed municipalities, as shown in Figure 2 above. This modeling approach implements, within a local linear regression framework (R²), the distance decay effect following the effect of the first law of geography, that is, the regression coefficients are not stationary, but estimated individually for each spatial analysis unit (municipalities) and a smoothing effect is applied, considering a neighbor matrix^(31)^.

To create the choropleth maps, the cartographic database of the state of Minas Gerais, including its respective municipalities and macro-regions, was used. It was obtained free of charge from the IBGE website and processed using ArcGIS 10.8 software.

This study uses public domain data with unrestricted access, without any identification of the individuals participating in the investigation. Therefore, submission for review by the Research Ethics Committee (REC) was not required.

## Results

**Figure 3 -:**
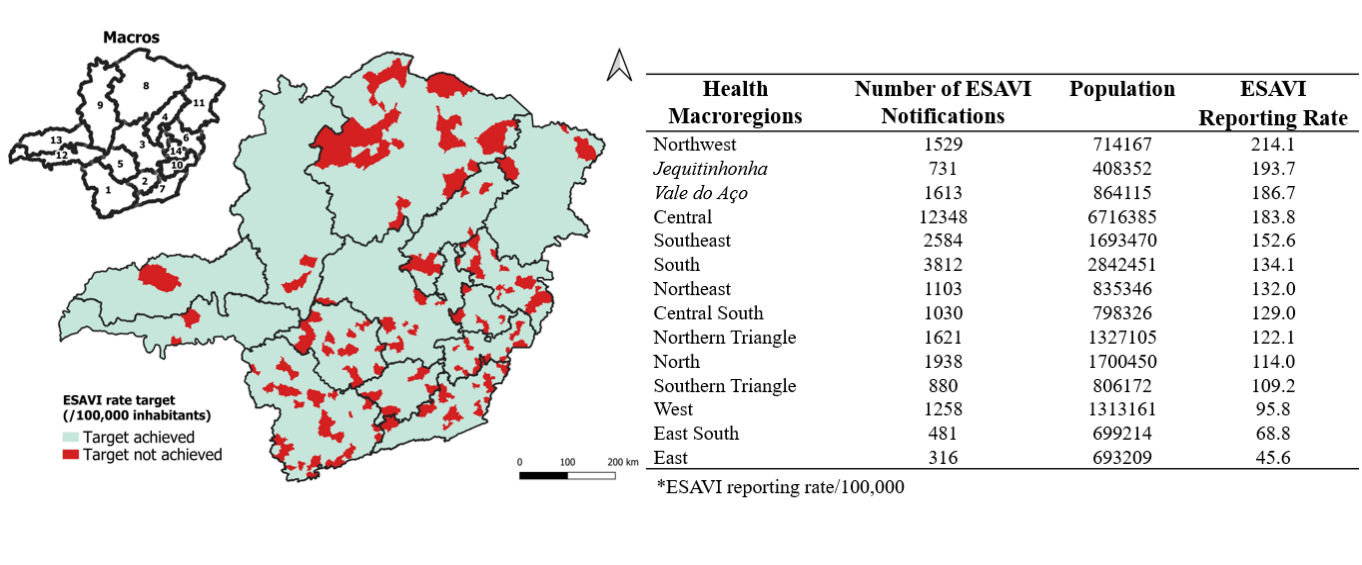
Achievement of the target for reporting rates of events supposedly attributable to vaccination or immunization against COVID-19. Minas Gerais, Brazil, 2021

Of the 853 municipalities in the state of Minas Gerais, 20.3% (n=173) did not reach the recommended target of a minimum number of 10 ESAVI notifications (reporting rate). The municipalities in the Northwest, Jequitinhonha and Vale do Aço macro-regions had the highest reporting rates in the state. The municipalities in the East, East South and West macro-regions had the lowest rates (Figure 3). There is a significant heterogeneity in the achievement of the ESAVI surveillance target among the municipalities of Minas Gerais.

**Figure 4 -:**
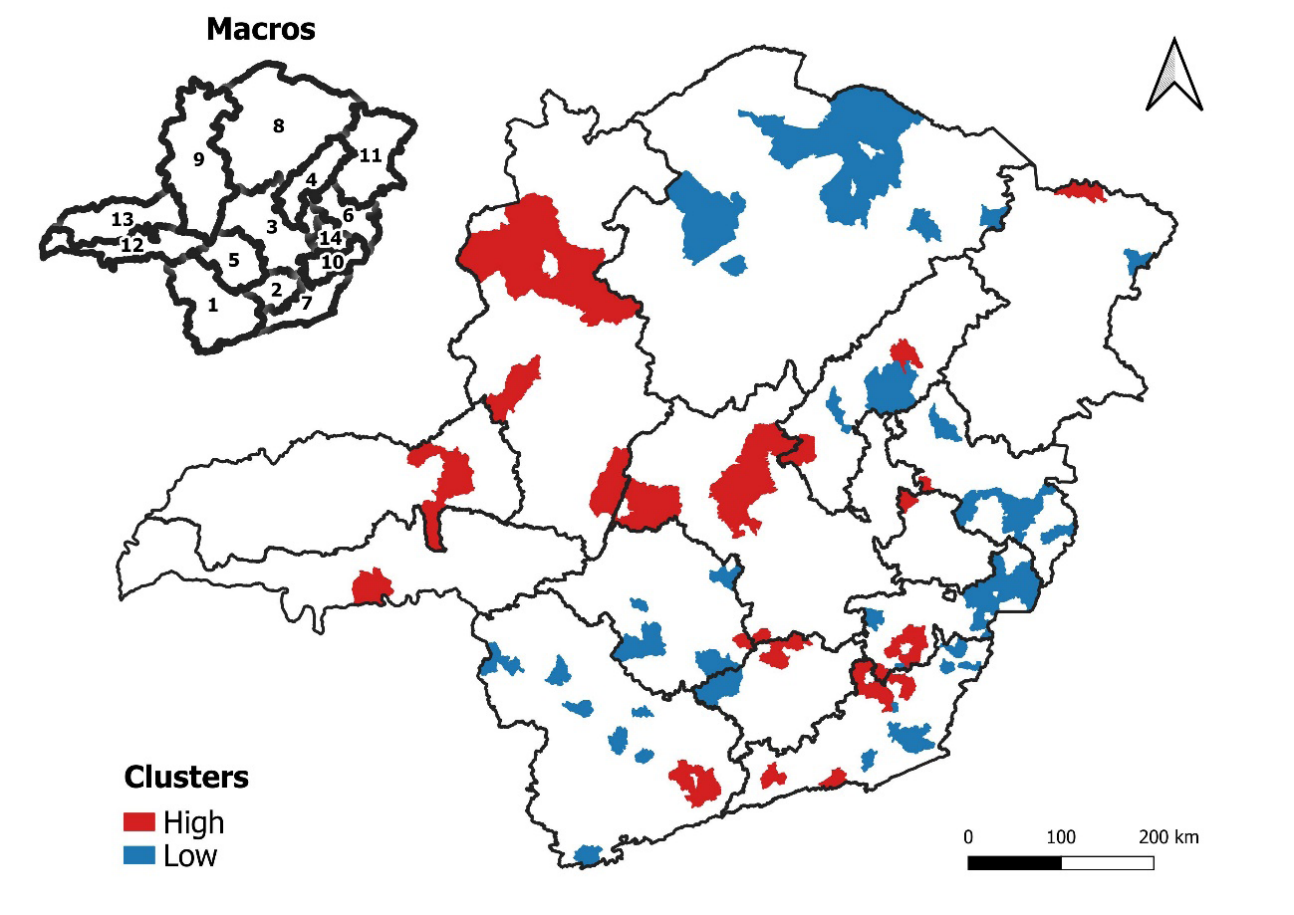
Spatial clusters of reporting rates of events supposedly attributable to vaccination or immunization against COVID-19. Minas Gerais, Brazil, 2021

The Getis-Ord Gi* analysis identified a heterogeneous distribution of ESAVI reporting rates resulting from COVID-19 vaccines throughout the state of Minas Gerais. The presence of two large clusters with low reporting rates, identified as cold areas, in the North macro-region, stands out. Clusters with a high reporting rate, considered hot areas, include the Northwest, Central and Northern Triangle macroregions (Figure 4).

Linear regression, considering the ESAVI reporting rate and the number of nursing professionals per inhabitant (regression coefficient= 0.644), suggests that there is a positive association between the variables, that is, as the number of nursing professionals increases, the ESAVI reporting rate also tends to increase, and the p-value was < 0.01. Another variable highlighted was the percentage of families living in rural areas, which presented a negative coefficient (-0.013), and it indicates that, as there is an increase in families living in rural areas, there is a decrease in the ESAVI reporting rate, and the p-value was < 0.01.

The Akaike Information Criterion (AIC) value was calculated as 3617.779, which provides a measure of the quality of the model, considering the balance between fit and complexity. The lower the AIC value, the better the model fit. Furthermore, the coefficient of determination (R²) was calculated as 0.03, indicating that approximately 3% of the variation in the ESAVI reporting rate can be explained by the variables included in the model (Akaike Information Criterion: 3617.779 and R²: 0.03).

**Figure 5 -:**
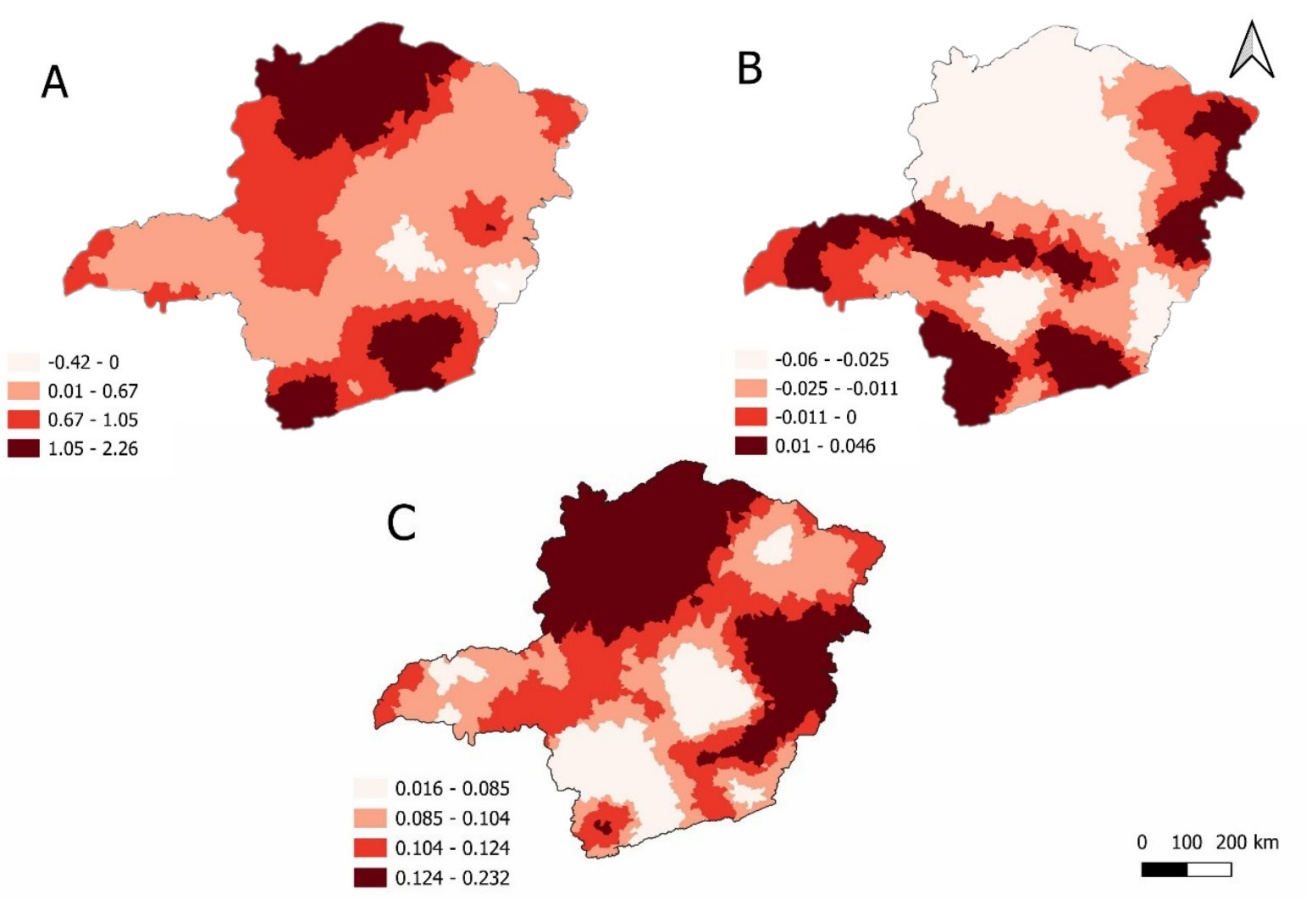
Geographically weighted spatial regression (GWR) analysis for the reporting rate of events supposedly attributable to vaccination or immunization against COVID-19 in Minas Gerais municipalities. Minas Gerais, Brazil, 2021

The association of the ESAVI reporting rate in relation to the variables number of nursing professionals per inhabitant and percentage of families living in rural areas is distributed heterogeneously throughout the state of Minas Gerais, that is, each variable has different associations (coefficient value) according to the regions (Figure 5).

When analyzing the Geographically weighted regression, it was observed that the ESAVI reporting rate has a positive association considering the number of nursing professionals per inhabitant (variable A) in the North and South regions of the state.

Regarding the regression coefficient of the percentage of families living in rural areas (variable B), a negative association was identified in a large part of the state, with the lowest value located in a vast area in the North region, followed by the West region.

The Local R² (variable C) demonstrates the explanatory capacity of the occurrence of the ESAVI reporting rate resulting from vaccines against COVID-19 in Minas Gerais. The explanatory capacity of the association between the explanatory variables and the ESAVI reporting rate is greater in the North and South regions of the state and lower in the Central region.

*A = Number of nursing professionals per inhabitant; ^†^B = Percentage of families living in rural areas; ^‡^C = Local R²

## Discussion

In Minas Gerais, clusters with low reporting rates, or even without any notification, were observed regarding the surveillance of ESAVI resulting from vaccines against COVID-19. Furthermore, it was found that, despite all macro-regions meeting the reporting rate target, 20.3% of their municipalities did not achieve this objective. Two factors were associated with reporting rates: the number of nursing professionals per inhabitant and the percentage of families living in rural areas. The distribution of these variables associated with the ESAVI reporting rate was heterogeneous throughout the state.

Studies indicate that the heterogeneity in ESAVI reporting rates is related to the geographic location, the level of awareness and training of health professionals, as well as the population’s knowledge about adverse event reporting^(15,36-37)^. These factors reinforce the importance of educational strategies and raising awareness among the population, combined with the training of health professionals, to promote a robust system of surveillance of adverse events and, consequently, safety and confidence in vaccination practices. Other factors associated with ESAVI include increased adverse event reporting in the early years of new vaccine licensing and increased reporting following the release of a specific adverse event^(15)^.

Regarding geographical location, Minas Gerais has a vast territorial extension and regions with different socioeconomic developments^(38)^. The Northwest and Vale do Aço regions, which have the best socioeconomic indicators, showed the highest ESAVI reporting rates for COVID-19. Meanwhile, the *Jequitinhonha* region, despite being notable for its low socioeconomic indicators, also reported high ESAVI reporting rates. Clusters of low reporting rates were identified in the Northern region of the state. The Northern region of Minas Gerais, when compared to other regions of the state, occupies a disadvantageous position due to its low socioeconomic indicators, such as average per capita income and social vulnerability^(38-39)^.

An international research reinforced that more developed regions tend to report more ESAVI than less developed regions^(17)^. It is assumed that more developed regions have a more adequate healthcare infrastructure, which may facilitate access to vaccination, increase vaccination coverage, and consequently raise the reporting of adverse events. Additionally, these regions are likely to have more effective communication strategies, capable of informing and encouraging the population to report post-vaccination events. On the other hand, despite the socioeconomic disparities in the *Jequitinhonha* region, it is possible to assume that the region has shown greater awareness and engagement of the population regarding the reporting of COVID-19 adverse events or even more intense efforts in raising awareness and post-vaccination monitoring.

Heterogeneity in reporting rates is also observed across Brazil. Only 46% of Brazilian municipalities made at least one ESAVI notification, and the majority of silent municipalities were concentrated in the North (23.6%) and Northeast (38.6%) regions of the country^(9)^. Furthermore, the lowest incidences of reported adverse events were observed in the North region of the country^(9,40)^.

This event is present beyond Brazilian borders. A study conducted in 194 countries demonstrated that there is great heterogeneity in the reporting of ESAVI between regions and countries^(15)^. The results of this study showed that only 64% of countries reported ESAVI through the World Health Organization Joint Reporting Form on Immunization. Among countries in the Americas and Africa, 83% and 51% made at least 1 report, respectively^(15)^. The authors also noted the existence of silent countries. The results of our research also identified some municipalities in Minas Gerais that share this silence regarding ESAVI notifications.

Regional disparities, both geographic and economic, reinforce the importance of spatial analyses to identify silent reporting areas and create interventions targeted at the region, as these differences can compromise the actions and services provided by municipalities^(38-39,41-44)^. It is important to consider that each location is at different stages of development, which is why the primary purpose of the ESAVI reporting rate is to establish a standard for evaluating the progress of the performance of ESAVI surveillance systems^(13-14)^.

Regardless of the factors that contribute to differences in ESAVI reporting requirements between countries and regions, ESAVI surveillance is characterized by a high degree of underreporting^(43)^. This lack of data constitutes a significant obstacle to formulating effective strategies in silent regions^(44)^.

In passive surveillance, the lack of notifications of ESAVI, for example, does not necessarily imply the absence of these events. Underreporting, understood as the lack of registration or official notification of cases that actually occurred, results in an underestimation of the real incidence or prevalence figures of the disease and/or event^(45)^. It is clear that underreporting is not only linked to regional differences (geographical, cultural and economic aspects); it can be attributed to other factors, such as the lack of continuing education of professionals, lack of human resources, lack of nursing supervision, work overload, lack of motivation and lack of vaccination screening^(7,9,46-47)^. These factors are attributed to the capacity and professional training to work in immunization services.

In the Brazilian context, the nursing team is primarily responsible for activities in the vaccination room. Geographically weighted regression (GWR) indicated a positive association between the ESAVI reporting rate and the number of nursing professionals per inhabitant. The literature emphasizes that the actions adopted by these professionals play a crucial role in preventing underreporting of ESAVI^(20-21,24)^. Carrying out vaccination screening, focusing on guidance on the vaccines administered and possible ESAVI caused by them, is a conduct that increases notifications, confidence in immunobiologicals and progress in passive surveillance^(20-21,24,48)^.

International studies have also highlighted that the conduct of health professionals who carry out vaccination screening increases the reporting of adverse events and, consequently, their incidence^(37,48-50)^. However, a study conducted in Mysuru, India, found that more than half (73.37%) of professionals who attended an ESAVI did not report it^(50)^. There is a presence of underreporting of the event, associated with professionals’ doubts about the events that should be reported, with a focus on serious and less frequent events^(11)^.

In addition to doubts regarding ESAVI, professionals are afraid to report due to fear of reprimand, lack of knowledge of the vaccinated person, work overload and immunization error^(22-24,51)^. This reinforces the need for training professionals in ESAVI surveillance, including the use of vaccine screening for community awareness, continuing education and supervision^(29-30)^.

Finally, underreporting of ESAVI can also occur due to technological issues such as software failures, problems in technological infrastructure, limited connectivity, lack of interoperability, overload and standardization of health information systems (HIS), which can make data collection and transmission difficult^(9,52-54)^. These technological issues experienced in services can intensify the underreporting of ESAVI and the quality of information. Due to the pandemic, for example, most systems had to deal with a high volume of data, which directly influenced the incompleteness of data and the inclusion of errors, which can result in underreporting^(54)^.

Another factor associated with the ESAVI reporting rate was the percentage of families living in rural areas. The literature shows that residents of these locations face considerable challenges in accessing vaccination rooms. The distance between residences and healthcare services, difficulties accessing public transportation, poor road maintenance, and the need to travel long distances to reach healthcare facilities are geographical barriers that directly impact the vaccination process^(7,25,56-57)^. Consequently, these factors hinder the monitoring of ESAVI cases, resulting in a reduction in the reporting of such events.

This study is based on secondary data, which implies some limitations that must be considered. The quality of the results is directly linked to the accuracy and reliability of the original data, and may be affected by possible inconsistencies or underreporting. Furthermore, the insertion of notifications into *e-SUS Notifica* is carried out by several professionals, which may generate different fillings. Therefore, when interpreting the results of this study, it is crucial to consider these limitations and assess how they may influence the instructions and applicability of the results in different contexts and settings.

However, the findings demonstrate the potential of the spatial analysis technique, as it identified the health macroregions of Minas Gerais that require priority interventions for the progress of ESAVI surveillance. And the strength of the study focuses on the advancement of scientific knowledge by providing a detailed analysis of the spatial distribution of ESAVI notifications against COVID-19 and associated factors. By applying advanced spatial analysis methods, such as Getis-Ord Gi* analysis and Geographically weighted regression, the study identified relevant geographic patterns in reporting rates and revealed regional disparities, highlighting areas with low adherence to event reporting. The importance of factors such as the number of nursing professionals per inhabitant and the percentage of families living in rural areas in influencing these rates is highlighted, contributing to a deeper understanding of the social and structural determinants that affect the reporting of events.

## Conclusion

The analysis of the spatial distribution of ESAVI notifications in the state identified silent municipalities that did not meet the minimum ESAVI notification value. The presence of clusters points to the need to implement integrated strategies, adapted to the particularities of each region, to improve surveillance of the safety of vaccines against COVID-19 in every Brazilian state.

The results of this article are useful for optimizing surveillance strategies. The Regional Health Superintendencies/Management Offices can use the data provided to identify areas with the highest and lowest ESAVI reporting rates, allocate additional resources, closely monitor vaccination practices, and implement targeted educational campaigns in these regions.

Furthermore, the analysis of spatial heterogeneity revealed socioeconomic patterns associated with these occurrences, allowing more targeted strategies for vulnerable groups. Inter-institutional collaboration is also possible, facilitating the exchange of best practices between regions. In summary, the data provided a solid basis for informed decision-making, strengthening the capacity of health superintendencies to promote safety and efficacy in vaccine monitoring.
