# Supplementary material for: Geopharmacosurveillance of reporting rates of events supposedly attributable to vaccination or immunization against COVID-19
Source: Rev Lat Am Enfermagem. 2025 May 2;33:e4539. doi: 10.1590/1518-8345.7509.4539 (PMC12052311; doi:10.1590/1518-8345.7509.4539)
Supplement: Translation

# Translation: Geofarmacovigilância das taxas de notificação de eventos supostamente atribuíveis à vacinação ou imunização contra a COVID-19*

**DOI:** 10.1590/1518-8345.7509.4540

**Authors:** Thays Cristina Pereira Barbosa, Gabriela Lourença Martins do Nascimento, Luiz Henrique Arroyo, Ricardo Alexandre Arcêncio, Valéria Conceição de Oliveira, Eliete Albano de Azevedo Guimarães

**Affiliations:** 1 Universidade Federal de São João del-Rei, Campus Centro-Oeste Dona Lindu, Divinópolis, MG, Brasil; 2 Bolsista da Coordenação de Aperfeiçoamento de Pessoal de Nível Superior (CNPq), Brasil; 3 Ministério da Saúde, Secretaria de Vigilância em Saúde, Brasília, DF, Brasil; 4 Universidade de São Paulo, Escola de Enfermagem de Ribeirão Preto, Centro Colaborador da OPAS/OMS para o Desenvolvimento da Pesquisa em Enfermagem, Ribeirão Preto, SP, Brasil

**Keywords:** Vacinas, Eventos Adversos, COVID-19, Vigilância em Saúde Pública, Análise Espacial, Estudos Ecológicos

## Abstract

analisar a distribuição espacial da taxa de notificação de eventos supostamente atribuíveis à vacinação ou imunização contra a COVID-19 e fatores associados ao cumprimento da meta preconizada, conforme o Plano de Ação Global para Vacinas.

estudo ecológico que considerou as notificações dos 853 municípios do estado de Minas Gerais. Foram analisadas 34.027 notificações, presentes no e-SUS Notifica. Realizou-se Getis-Ord Gi* e Regressão geograficamente ponderada para identificar agrupamentos espaciais, cumprimento de no mínimo 10 notificações e fatores correlacionados com a distribuição espacial.

observou-se distribuição heterogênea das taxas de notificação em todo o estado. 20,3% dos municípios não cumpriram a meta preconizada de notificações. Os municípios das macrorregiões Noroeste, Jequitinhonha e Vale do Aço tiveram as maiores taxas de notificação do estado. Já os municípios das macrorregiões Leste, Leste Sul e Oeste tiveram as menores taxas. O número de profissionais de enfermagem por habitante (coeficiente de regressão= 0,644; p< 0,01) e o percentual de famílias residentes em área rural (coeficiente de regressão= -0,013; p< 0,01) estiveram associados às taxas de notificação.

a presença de aglomerados de baixas notificações aponta a necessidade de implementar estratégias integradas e adaptadas às particularidades de cada região, para aprimorar a vigilância das notificações de eventos.

## Destaques

**(1)** Heterogeneidades espaciais das taxas de notificação do estado de Minas Gerais. **(2)** Identificação de áreas que necessitam de intervenções prioritárias de vigilância. **(3)** Necessidade de implementar ações locais para reduzir subnotificações de ESAVI. **(4)** Identificação de fatores associados à taxa de notificação de ESAVI. **(5)** Contribuição para políticas de saúde alinhadas a metas globais.

## Introdução

A Organização Mundial da Saúde (OMS) reconhece a imunização como uma das principais metas da agenda 2030, estando presente no terceiro objetivo como forma de assegurar uma vida saudável e promover o bem-estar para todos, em todas as idades^(1)^. A vacinação proporciona proteção contra uma gama diversificada de doenças infecciosas, entre elas a COVID-19. Embora seja referida como uma das mais seguras e eficazes intervenções preventivas em saúde, a vacinação não está isenta de Eventos Supostamente Atribuíveis à Vacinação ou Imunização (ESAVI)^(2-3)^.

Um ESAVI é qualquer sintoma laboratorial anormal ou doença, desfavorável e indesejado, cuja sua ocorrência se dá após a vacinação, e que não possui precisamente uma relação causal com o uso de uma vacina e o processo de imunização, podendo ser considerado um evento adverso grave (EAG) ou um evento adverso não grave (EANG)^(3)^.

A vigilância contínua de ESAVI é necessária para identificar e mitigar riscos relacionados às vacinas e práticas de vacinação^(4)^. Para isso, a notificação dos eventos desempenha um papel crucial, sendo uma prática essencial na vigilância^(5)^. Essa notificação permite a investigação detalhada dos casos, facilitando a compreensão da ocorrência, causalidade e evolução dos eventos. Além disso, possibilita a identificação de riscos, grupos populacionais mais vulneráveis e as realidades contextuais em que estão inseridos, considerando fatores institucionais/organizacionais, ambientais, políticos e socioeconômicos^(6-11)^.

O período analisado neste estudo, de janeiro a dezembro de 2021, foi marcado pela maior campanha de vacinação da história do Brasil, promovida pelo Ministério da Saúde. Ao longo desse ano, mais de 350 milhões de doses da vacina contra a COVID-19 foram distribuídas em todo o país. Essa mobilização massiva resultou em um aumento expressivo no volume de notificações, reflexo da alta procura pelo imunizante e da intensificação das ações de vacinação^(12)^. Este contexto ressalta a importância da notificação como um indicador de desempenho da vigilância, permitindo a identificação de padrões e lacunas no sistema de registro e monitoramento de eventos adversos (erros de digitação, incompletude de campos e casos de subnotificações)^(8-11,13-14)^.

Dada a significância da vigilância de ESAVI, o Plano de Ação Global para Vacinas (GVAP) estabeleceu a taxa de notificação como um indicador de desempenho para avaliar o progresso da vigilância passiva da segurança das vacinas^(13-14)^. A taxa de notificação analisa o número de notificações de ESAVI em uma dada região, com base na sua população, e o padrão consiste em que a região deve atingir no mínimo 10 notificações^(15-16)^. Os resultados dessa medida são úteis para análises comparativas entre países e regiões e podem apoiar a definição de metas para a melhoria progressiva da vigilância de ESAVI, adaptadas às particularidades de cada localidade^(16)^.

Um estudo comparativo das tendências das taxas de notificação de ESAVI global, entre os anos de 2000 a 2015, observou oscilações positivas nas taxas, indicando que o número de países com taxas de notificação de ESAVI superiores a 10 aumentou de 8 (4%) em 2000 para 81 (42%) em 2015^(15)^. Seguindo na mesma vertente, uma pesquisa identificou um progresso global na proporção de países que realizaram a notificação de ESAVI entre 2010 e 2019, aumentando de 41,2% para 56,2%^(17)^. Ademais, um estudo realizado nos Estados Unidos conduziu uma análise da taxa de notificação com base na população, revelando uma tendência crescente nas notificações de ESAVI no país^(16)^.

No Brasil, não foram identificados estudos que abordassem a taxa de notificação para analisar o desempenho dos serviços de imunização na vigilância passiva dos ESAVI. Em se tratando das notificações de ESAVI das vacinas contra a COVID-19 no país, estas são realizadas por meio do e-SUS Notifica, *software* que utiliza uma ficha de notificação/investigação, após a procura espontânea do identificador do evento^(18-19)^. O monitoramento das informações sobre os ESAVI permite a identificação de notificações, o acompanhamento de casos e o aprimoramento da qualidade das informações^(8-11)^.

Contudo, a subnotificação dos ESAVI tem sido um desafio preocupante, evidenciando a necessidade de aprimoramento dos sistemas de vigilância nos municípios brasileiros^(9,20)^. A subnotificação pode ser atribuída a diversos fatores, tais como a insuficiência de infraestrutura de saúde, a conduta e falta de capacitação adequada dos profissionais, deficiência de recursos humanos, falta de insumos, falta de supervisão de enfermagem, sobrecarga de trabalho, desmotivação, não conscientização da população, e aspectos geográficos, culturais e econômicos da região^(21-26)^.

Diante desse cenário, tornam-se indispensáveis uma atenção singular e um planejamento estratégico condizente com as características de cada localidade brasileira para o avanço da vigilância passiva da segurança das vacinas.

Esse tipo de vigilância busca compreender as interações entre medicamentos, meio ambiente e saúde humana, utilizando técnicas espaciais para monitorar e identificar áreas de potencial risco e implementar medidas preventivas e corretivas direcionadas a determinadas regiões. A geofarmacovigilância de vacinas realiza o monitoramento espacial de ESAVI ou de qualquer outro problema relacionado à vacinação ou imunização, auxiliando na detecção de possíveis tendências, clusters ou áreas com maior incidência do evento^(27)^.

O presente estudo analisou a distribuição espacial da taxa de notificação de eventos supostamente atribuíveis à vacinação ou imunização contra a COVID-19 e fatores associados ao cumprimento da meta preconizada, conforme o Plano de Ação Global para Vacinas.

## Método

### Delineamento do estudo

Trata-se de um estudo ecológico, norteado pela ferramenta *SQUIRE* 2.0 (*Standards for Quality Improvement Reporting Excellence*).

### Cenário do estudo

O estudo foi realizado em Minas Gerais, o segundo estado mais populoso do Brasil, com uma população estimada de 21.411.923 milhões de habitantes, um grau de urbanização de 85,29%, um Índice de Desenvolvimento Humano (IDH) médio alto, e uma economia diversificada^(28)^. A escolha de Minas Gerais para a realização deste estudo é justificada pela sua grande diversidade demográfica, social e econômica, além de abrigar o maior número de municípios do país, o que oferece um cenário rico e complexo para análise. Ademais, a parceria entre a Secretaria de Estado de Saúde (SES-MG) e a Universidade Federal de São João del-Rei (UFSJ), por meio de projetos colaborativos, garante maior aplicabilidade e impacto dos resultados.

Os 853 municípios estão distribuídos em 14 regiões de saúde: Sul (3101), Centro Sul (3102), Centro (3103), Jequitinhonha (3104), Oeste (3105), Leste (3106), Sudeste (3107), Norte (3108), Noroeste (3109), Leste Sul (3110), Nordeste (3111), Triângulo do Sul (3112), Triângulo do Norte (3113) e Vale do Aço (3114) (Figura 1). Estas, por sua vez, englobam 89 microrregiões de saúde e 28 Superintendências/Gerências Regionais de Saúde^(28-29)^.

Para este estudo, foram estabelecidas como unidades territoriais de análise os 853 municípios das 14 macrorregiões do estado (Figura 1).

**Figura 1 -:** Regiões de saúde do estado de Minas Gerais, Brasil, 2022

### População e coleta de dados

Foram analisadas as notificações de ESAVI das vacinas contra a COVID-19, com e sem encerramento, disponíveis na plataforma eletrônica do Departamento de Informática do Sistema Único de Saúde (DATASUS), sistema e-SUS Notifica^(19)^, entre janeiro e dezembro de 2021. A escolha pela vacinação contra a COVID-19 foi motivada pela relevância e urgência imposta pela pandemia, além de ser um evento recente e de alto impacto na saúde pública. Foram incluídas nesta pesquisa todas as 34.027 notificações de ESAVI, e excluídos os erros de imunização (n= 2663).

A variável desfecho foi o número de notificações de ESAVI, com e sem encerramento. A notificação de ESAVI é utilizada para detectar riscos relacionados às vacinas ou práticas de vacinação, utilizando-se da ficha de notificação/investigação de eventos adversos^(4)^. Neste estudo, calculou-se a taxa de notificação de ESAVI, que consiste no número de notificações de ESAVI dividido pela população em uma dada região, multiplicado por 100.000. O padrão utilizado para avaliar a taxa de notificação foi o número mínimo de 10 notificações por 100.000 habitantes^(13-16)^.

Para identificar os fatores associados ao cumprimento das taxas de notificação de ESAVI, foram selecionadas variáveis de exposição, provenientes do Cadastro Nacional dos Estabelecimentos de Saúde do Brasil (CNES), da base do Instituto Brasileiro de Geografia e Estatística (IBGE), e da Fundação João Pinheiro (FJP), que foram incluídas nas análises de regressão logística (Figura 2).

**Figura 2 -:** Descrição das variáveis de exposição consideradas para o estudo. Minas Gerais, Brasil, 2023

### Tratamento e análise de dados

Inicialmente, na fase exploratória, foi utilizado o *software Microsoft Office Excel*, versão 2016, para verificar a consistência e a qualidade dos registros das notificações de ESAVI.

Além disso, utilizou-se a taxa de notificação de ESAVI (número de notificações de ESAVI dividido pela população de uma dada região, multiplicado por 100.000) para criar os mapas referentes às análises Getis-Ord Gi* e Regressão geograficamente ponderada (*Geographically weighted regression* - GWR)^(30-31)^.

A ferramenta utilizada para determinar a distribuição das taxas de notificação de ESAVI no espaço foi o *software GeoDa 1.20.* Trata-se de um instrumento de *software* livre e de código projetado para a realização de métodos de análise exploratória de dados espaciais (ESDA), como estatísticas de autocorrelação espacial. Ele também disponibiliza um módulo estatístico para a análise Getis-Ord Gi*. E a análise GWR foi realizada pelo *software* livre *R 4.3,* para computação estatística e gráficos^(32-34)^. Para todas as análises espaciais foi utilizada a matriz de vizinhança do tipo rainha com 1 ordem. Foram considerados todos os municípios que fazem fronteira direta e em todas as direções.

A análise Getis-Ord Gi* foi utilizada para identificar os aglomerados das taxas de notificação de ESAVI decorrentes das vacinas anti-COVID-19. A estatística consiste na razão da média ponderada dos valores das localidades vizinhas para a soma de todos os valores, incluindo o valor da localidade específica que é analisada em uma janela analítica. Dessa forma, valores acima da média são classificados como áreas de alta ocorrência, enquanto valores abaixo da média sugerem baixa ocorrência. Essa análise resulta em duas classificações: uma para municípios com valores elevados, estatisticamente significativos (área de alta ocorrência/área quente - *Hotspot*) e outro para municípios com valores baixos, também estatisticamente significativos (área de baixa ocorrência/área fria - *Coldspot)* ^(31,35)^.

Para identificar os fatores associados à taxa de notificação de ESAVI, foi elaborada uma regressão geograficamente ponderada, considerando variáveis explicativas que caracterizassem os municípios analisados (Figura 2). Esta abordagem de modelagem implementa, dentro de uma estrutura de regressão linear local (R²), o efeito de decaimento de distância, seguindo o efeito da primeira lei da geografia, ou seja, os coeficientes da regressão não são estacionários, mas estimados individualmente para cada unidade de análise espacial (municípios), e um efeito suavizador deste é aplicado, considerando uma matriz de vizinhos^(31)^.

Para elaborar os mapas coropléticos, foi utilizada a base cartográfica do estado de Minas Gerais, de seus respectivos municípios e macrorregiões, obtida gratuitamente no site do IBGE e processada por meio do *software* ArcGIS 10.8*.*

O presente estudo utilizou dados de domínio público e de acesso irrestrito, sem qualquer identificação dos indivíduos participantes da investigação. Portanto, não foi necessária a submissão à apreciação do Comitê de Ética em Pesquisa (CEP).

## Resultados

Dos 853 municípios do estado de Minas Gerais, 20,3% (n= 173) não alcançaram a meta preconizada de número mínimo de 10 notificações de ESAVI (taxa de notificação). Os municípios das macrorregiões Noroeste, Jequitinhonha e Vale do Aço tiveram as maiores taxas de notificação do estado. Já os municípios das macrorregiões Leste, Leste Sul e Oeste tiveram as menores taxas (Figura 3). Nota-se grande heterogeneidade no cumprimento da meta de vigilância de ESAVI entre os municípios de Minas Gerais.

Identificou-se na análise Getis-Ord Gi* uma distribuição heterogênea das taxas de notificação de ESAVI decorrentes de vacinas contra a COVID-19 em todo o estado de Minas Gerais. Destaca-se a presença de dois grandes aglomerados com baixa taxa de notificação, identificados como áreas frias, na macrorregião Norte. Os aglomerados com alta taxa de notificação, considerados áreas quentes, incluem as macrorregiões Noroeste, Centro e Triângulo do Norte (Figura 4).

**Figura 3 -:** Cumprimento da meta das taxas de notificação de eventos supostamente atribuíveis à vacinação ou imunização contra a COVID-19. Minas Gerais, Brasil, 2021

**Figura 4 -:** Aglomerados espaciais das taxas de notificação de eventos supostamente atribuíveis à vacinação ou imunização contra a COVID-19. Minas Gerais, Brasil, 2021

A regressão linear, considerando a taxa de notificação de ESAVI e o número de profissionais de enfermagem por habitante (coeficiente da regressão= 0,644), sugere que há uma associação positiva entre as variáveis, ou seja, à medida que o número de profissionais de enfermagem aumenta, a taxa de notificação de ESAVI também tende a aumentar, e o valor de p foi < 0,01. Outra variável em destaque foi o percentual de famílias residentes em área rural, que apresentou um coeficiente negativo (-0,013), o que indica que, à medida que há um aumento de famílias residentes em áreas rurais, há uma diminuição na taxa de notificação de ESAVI, e o valor de p foi < 0,01.

O valor do Critério de Informação de Akaike (AIC) foi calculado como 3617.779, o que fornece uma medida da qualidade do modelo, considerando o equilíbrio entre ajuste e complexidade. Quanto menor o valor do AIC, melhor é o ajuste do modelo. Além disso, o coeficiente de determinação (R²) foi calculado como 0.03, indicando que aproximadamente 3% da variação na taxa de notificação de ESAVI pode ser explicada pelas variáveis incluídas no modelo (*Akaike Information Criterion*: 3617.779 e R^2^: 0.03).

A associação da taxa de notificação de ESAVI em relação às variáveis número de profissionais de enfermagem por habitante e percentual de famílias residentes em área rural está distribuída de forma heterogênea em todo o estado de Minas Gerais, ou seja, cada variável tem diferentes associações (valor de coeficiente) segundo as regiões (Figura 5).

Ao analisar a regressão geograficamente ponderada, observou-se que a taxa de notificação de ESAVI tem associação positiva, considerando o número de profissionais de enfermagem por habitante (variável A), nas regiões Norte e Sul do estado.

Com relação ao coeficiente de regressão do percentual de famílias residentes em área rural (variável B), identificou-se associação negativa em grande parte do estado, sendo o menor valor localizado em uma vasta área na região Norte, seguida pela região Oeste.

O R² local (variável C) demonstra a capacidade de explicação da ocorrência da taxa de notificação de ESAVI decorrente de vacinas contra a COVID-19 em Minas Gerais. A capacidade de explicação da associação entre as variáveis explicativas e a taxa de notificação de ESAVI é maior nas regiões Norte e Sul do estado e menor na região Centro.

**Figura 5 -:** Análise de regressão espacial geograficamente ponderada (GWR) para a taxa de notificação de eventos supostamente atribuíveis à vacinação ou imunização contra a COVID-19 nos municípios mineiros. Minas Gerais, Brasil, 2021

*A = Número de profissionais de enfermagem por habitante; ^†^B = Percentual de famílias residentes em área rural; ^‡^C = R^2^ local

## Discussão

Em Minas Gerais, foram observados aglomerados com baixas taxas de notificação, ou até mesmo sem nenhuma notificação, com relação à vigilância dos ESAVI decorrentes das vacinas contra a COVID-19. Além disso, constatou-se que, apesar de todas as macrorregiões cumprirem a meta da taxa de notificação, 20,3% de seus municípios não atingiram esse objetivo. Dois fatores estiveram associados às taxas de notificação: o número de profissionais da enfermagem por habitante e o percentual de famílias residentes em área rural. A distribuição dessas variáveis associadas à taxa de notificação de ESAVI se apresentou de forma heterogênea em todo o estado.

Estudos indicam que a heterogeneidade nas taxas de notificação de ESAVI está relacionada à localização geográfica, ao nível de sensibilização e capacitação dos profissionais de saúde, bem como ao conhecimento da população sobre a notificação de eventos adversos^(15,36-37)^. Esses fatores reforçam a importância de estratégias educativas e de conscientização da população, aliadas à capacitação dos profissionais de saúde, para promover um sistema robusto de vigilância dos eventos adversos e, consequentemente, a segurança e a confiança nas práticas de vacinação. Outros fatores associados a ESAVI incluem o aumento nas notificações de eventos adversos nos primeiros anos de licenciamento de novas vacinas e o acréscimo de notificações após a divulgação de um evento adverso específico^(15)^.

Quanto à localização geográfica, Minas Gerais tem uma vasta extensão territorial e regiões com diferentes desenvolvimentos socioeconômicos^(38)^. As regiões Noroeste e Vale do Aço, que têm os melhores indicadores socioeconômicos, apresentaram as maiores taxas de notificação de ESAVI de COVID-19. Já a região do Jequitinhonha, apesar de se destacar por seus baixos indicadores socioeconômicos, também apresentou altas taxas de notificação de ESAVI. Os aglomerados de baixas taxas de notificação foram identificados na região Norte do estado. O Norte de Minas, quando comparado com outras regiões do estado, ocupa uma posição desvantajosa devido aos seus baixos indicadores socioeconômicos, como a renda média per capita e a vulnerabilidade social^(38-39)^.

Uma pesquisa internacional reforçou que regiões mais desenvolvidas tendem a notificar mais ESAVI que regiões menos desenvolvidas^(17)^. Pressupõe-se que regiões mais desenvolvidas possuam uma infraestrutura de saúde mais adequada, o que pode facilitar o acesso à vacinação, aumentar a cobertura vacinal e, consequentemente, elevar a notificação de eventos adversos. Além disso, essas regiões tendem a contar com estratégias de comunicação mais eficazes, capazes de esclarecer e incentivar a população a relatar ocorrências de eventos pós-vacinação. Por outro lado, apesar das disparidades socioeconômicas da região do Jequitinhonha, é possível pressupor que a região tenha apresentado uma maior conscientização e engajamento da população com relação à notificação de eventos adversos da COVID-19, ou até mesmo esforços mais intensos na sensibilização e acompanhamento pós-vacinação.

A heterogeneidade nas taxas de notificação também é observada no território brasileiro. Apenas 46% dos municípios brasileiros realizaram ao menos uma notificação de ESAVI, e a maioria dos municípios silenciosos concentrou-se nas regiões Norte (23,6%) e Nordeste (38,6%) do país^(9)^. Além disso, as menores incidências de eventos adversos notificados foram observadas na região Norte do país^(9,40)^.

Esse evento está presente para além das fronteiras brasileiras. Um estudo realizado com 194 países mostrou que existe uma grande heterogeneidade de notificação de ESAVI entre regiões e países^(15)^. Os resultados desse estudo apontaram que somente 64% dos países notificaram ESAVI por meio do Formulário de Notificação Conjunta sobre Imunização da Organização Mundial da Saúde (OMS). Entre os países da região das Américas e da África, 83% e 51% realizaram pelo menos uma notificação, respectivamente^(15)^. Os autores também observaram a existência de países silenciosos. Os resultados de nossa pesquisa também identificaram alguns municípios em Minas Gerais que compartilham desse silêncio com relação às notificações de ESAVI.

As disparidades regionais, tanto geográficas como econômicas, reforçam a importância de análises espaciais para a identificação de áreas silenciosas de notificação e a criação de intervenções direcionadas à região, já que essas diferenças podem comprometer as ações e os serviços disponibilizadas pelos municípios^(38-39,41-44)^. É importante considerar que cada localidade se encontra em diferentes estágios de desenvolvimento, razão pela qual o propósito primordial da taxa de notificação de ESAVI é estabelecer um padrão para avaliar o progresso do desempenho dos sistemas de vigilância de ESAVI^(13-14)^.

Independentemente dos fatores que contribuem para as diferenças nos requisitos de notificação de ESAVI entre países e regiões, a vigilância de ESAVI é caracterizada por um elevado grau de subnotificação^(43)^. Essa carência de dados constitui um obstáculo significativo para a formulação de estratégias eficazes nas regiões silenciosas^(44)^.

Na vigilância passiva, a falta de notificações de ESAVI, por exemplo, não implica, necessariamente, na ausência desses eventos. A subnotificação, entendida como a falta de registro ou notificação oficial de casos que efetivamente aconteceram, resulta em uma subestimação dos números reais de incidência ou prevalência da doença e/ou evento^(45)^. É notório que a subnotificação não está ligada somente às diferenças regionais (aspectos geográficos, culturais e econômicos); ela pode ser atribuída a outros fatores, como falta de educação continuada dos profissionais, deficiência de recursos humanos, ausência de supervisão de enfermagem, sobrecarga de trabalho, desmotivação e falta da triagem vacinal^(7,9,46-47)^. Fatores estes atribuídos à capacidade e formação profissional para atuação nos serviços de imunização.

No contexto brasileiro, é a equipe de enfermagem que detém a principal responsabilidade pelas atividades na sala de vacinação. A Regressão geograficamente ponderada (GWR) apontou uma associação positiva entre a taxa de notificação de ESAVI e o número de profissionais da enfermagem por habitante. A literatura enfatiza que as ações adotadas por esses profissionais desempenham um papel crucial na prevenção da subnotificação de ESAVI^(20-21,24)^. A realização da triagem vacinal, com foco na orientação sobre as vacinas administradas e possíveis ESAVI causados por elas, é uma conduta que aumenta as notificações, a confiança em relação aos imunobiológicos e o progresso da vigilância passiva^(20-21,24,48)^.

Estudos internacionais também destacaram que as condutas dos profissionais de saúde que realizam a triagem vacinal aumentam a notificação de eventos adversos e, consequentemente, sua incidência^(37,48-50)^. Entretanto, um estudo conduzido em Mysuru, na Índia, constatou que mais da metade (73,37%) dos profissionais que atenderam a um ESAVI não procederam à sua notificação^(50)^. Há uma presença de subnotificação do evento, associando-a a dúvidas dos profissionais sobre os eventos que deveriam ser notificados, com foco em eventos graves e menos frequentes^(11)^.

Além das dúvidas referentes aos ESAVI, os profissionais têm receios em notificar devido ao medo de repreensão, à falta de conhecimento do vacinado, à sobrecarga de trabalho e ao erro de imunização^(22-24,51)^. Isso reforça a necessidade de capacitação dos profissionais quanto à vigilância do ESAVI, incluindo o uso da triagem vacinal para a sensibilização da comunidade, educação continuada e supervisão^(29-30)^.

Por fim, a subnotificação de ESAVI também pode ocorrer devido a questões tecnológicas, como falhas nos *softwares*, problemas na infraestrutura tecnológica, conectividade limitada, falta de interoperabilidade, sobrecarga e padronização de sistemas de informação em saúde (SIS), o pode dificultar a coleta e a transmissão de dados^(9,52-54)^. Essas questões tecnológicas vivenciadas nos serviços podem intensificar a subnotificação dos ESAVI e a qualidade das informações. Em virtude da pandemia, por exemplo, grande parte dos sistemas tiveram que lidar com um elevado volume de dados, o que influenciou diretamente a incompletude dos dados e inclusão de erros, podendo resultar em subnotificações^(54)^.

Outro fator associado à taxa de notificação de ESAVI foi o percentual de famílias residentes em área rural. A literatura mostra que os moradores dessas localidades enfrentam desafios consideráveis no acesso às salas de vacinação. A distância entre as residências e os serviços de saúde, a dificuldade de acesso ao transporte público, a falta de manutenção nas estradas e a necessidade de percorrer longas distâncias até a unidade de saúde são barreiras geográficas que impactam diretamente o processo de vacinação^(7,25,56-57)^. Por conseguinte, tais fatores dificultam o monitoramento dos casos de ESAVI, resultando em uma redução das notificações desses eventos.

Esse estudo baseia-se em dados secundários, o que implica em algumas limitações que devem ser consideradas. A qualidade dos resultados está diretamente ligada à precisão e confiabilidade dos dados originais, podendo ser afetada por possíveis inconsistências ou subnotificações. Além disso, a inserção das notificações no e-SUS Notifica é realizada por vários profissionais, podendo gerar preenchimentos diferentes. Portanto, ao interpretar os resultados deste estudo, é crucial considerar estas limitações e avaliar como elas podem influenciar as instruções e a aplicabilidade dos resultados em diferentes contextos e cenários.

Contudo, os achados identificados demonstram o potencial da técnica de análise espacial, pois identificou as macrorregiões de saúde de Minas Gerais que necessitam de intervenções prioritárias para o progresso da vigilância de ESAVI. E a fortaleza do estudo incide no avanço do conhecimento científico ao fornecer uma análise detalhada da distribuição espacial das notificações de ESAVI contra a COVID-19 e fatores associados. Através da aplicação de métodos avançados de análise espacial, como a análise de Getis-Ord Gi* e a Regressão geograficamente ponderada, o estudo identificou padrões geográficos relevantes nas taxas de notificação e revela disparidades regionais, destacando áreas com baixa adesão à notificação de eventos. Destaca-se a importância de fatores como o número de profissionais de enfermagem por habitante e a porcentagem de famílias residentes em áreas rurais na influência dessas taxas, contribuindo para uma compreensão mais aprofundada dos determinantes sociais e estruturais que afetam a notificação de eventos.

## Conclusão

A análise da distribuição espacial das notificações de ESAVI no estado identificou municípios silenciosos que não atenderam ao valor mínimo de notificação de ESAVI. A presença de aglomerados aponta a necessidade de implementar estratégias integradas, adaptadas às particularidades de cada região, para aprimorar a vigilância da segurança das vacinas contra a COVID-19 em todo estado brasileiro.

Os resultados deste artigo são úteis para a otimização de estratégias de vigilância. As Superintendências/Gerências Regionais de Saúde podem utilizar os dados fornecidos para identificar as áreas com maiores e menores taxas de notificação de ESAVI, direcionar recursos adicionais, monitorar de perto as práticas de vacinação, e implementar campanhas educativas específicas nessas regiões.

Além disso, a análise da heterogeneidade espacial revelou padrões socioeconômicos associados a essas ocorrências, permitindo estratégias mais direcionadas para grupos vulneráveis. A colaboração interinstitucional também se torna possível, facilitando a troca de melhores práticas entre regiões. Em suma, os dados ofereceram uma base sólida para a tomada de decisões informadas, fortalecendo a capacidade das superintendências de saúde de promover a segurança e a eficácia no monitoramento das vacinas.

# Translation: Geofarmacovigilancia de las tasas de notificación de eventos supuestamente atribuibles a la vacunación o inmunización contra la COVID-19*

**DOI:** 10.1590/1518-8345.7509.4538

**Authors:** Thays Cristina Pereira Barbosa, Gabriela Lourença Martins do Nascimento, Luiz Henrique Arroyo, Ricardo Alexandre Arcêncio, Valéria Conceição de Oliveira, Eliete Albano de Azevedo Guimarães

**Affiliations:** 1 Universidade Federal de São João del-Rei, Campus Centro-Oeste Dona Lindu, Divinópolis, MG, Brasil; 2 Becaria de la Coordenação de Aperfeiçoamento de Pessoal de Nível Superior (CNPq), Brasil; 3 Ministério da Saúde, Secretaria de Vigilância em Saúde, Brasília, DF, Brasil; 4 Universidade de São Paulo, Escola de Enfermagem de Ribeirão Preto, Centro Colaborador de la OPS/OMS para el Desarrollo de la Investigación en Enfermería, Ribeirão Preto, SP, Brasil

**Keywords:** Vacunas, Evento Adverso, COVID-19, Vigilancia en Salud Pública, Análisis Espacial, Estudios Ecológicos

## Abstract

analizar la distribución espacial de la tasa de notificación de eventos supuestamente atribuibles a la vacunación o inmunización contra la COVID-19 y factores asociados al cumplimiento de la meta recomendada, según el Plan de Acción Mundial sobre Vacunas.

estudio ecológico que consideró notificaciones de 853 municipios del estado de Minas Gerais. Fueron analizadas 34.027 notificaciones presentes en *e-SUS Notifica*. Se realizaron Getis-Ord Gi* y Regresión geográficamente ponderada para identificar agrupaciones espaciales, cumplimiento de al menos 10 notificaciones y factores correlacionados con la distribución espacial.

se observó una distribución heterogénea de las tasas de notificación en todo el estado. El 20,3% de los municipios no cumplió con la meta de notificación recomendada. Los municipios de las macrorregiones Noroeste, Jequitinhonha y Vale do Aço tuvieron las mayores tasas de notificación del estado. Los municipios de las macrorregiones Este, Este Sur y Oeste tuvieron las tasas más bajas. El número de profesionales de enfermería por habitante (coeficiente de regresión= 0,644; p< 0,01) y el porcentaje de familias residentes en áreas rurales (coeficiente de regresión= -0,013; p< 0,01) se asociaron con las tasas de notificación.

la presencia de aglomerados de bajas notificaciones señala la necesidad de implementar estrategias integradas y adaptadas a las particularidades de cada región, para mejorar la vigilancia de las notificaciones de eventos.

## Destacados

**(1)** Heterogeneidades espaciales de las tasas de notificación del estado de Minas Gerais. **(2)** Identificación de áreas que requieren intervenciones prioritarias de vigilancia. **(3)** Necesidad de implementar acciones locales para reducir la subnotificación de ESAVI. **(4)** Identificación de factores asociados a la tasa de notificación de ESAVI. **(5)** Contribución a políticas de salud alineadas con metas globales.

## Introducción

La Organización Mundial de la Salud (OMS) reconoce la inmunización como una de las principales metas de la agenda 2030, estando presente en el tercer objetivo como una forma de garantizar una vida saludable y promover el bienestar para todos, en todas las edades^(1)^. La vacunación brinda protección contra una amplia gama de enfermedades infecciosas, incluida la COVID-19. Aunque se la refiere como una de las intervenciones preventivas de salud más seguras y efectivas, la vacunación no está exenta de Eventos Supuestamente Atribuibles a la Vacunación o Inmunización (ESAVI)^(2-3)^.

Un ESAVI es cualquier síntoma anormal de laboratorio o enfermedad, desfavorable y no deseado, que se presenta después de la vacunación, y que no tiene necesariamente una relación causal con el uso de una vacuna y el proceso de inmunización, pudiendo considerarse un evento adverso grave (EAG) o un evento adverso no grave (EANG)^(3)^.

Es necesaria una vigilancia continua de ESAVI para identificar y mitigar los riesgos relacionados con las vacunas y las prácticas de vacunación^(4)^. Para ello, la notificación de eventos juega un papel crucial, siendo una práctica esencial en la vigilancia^(5)^. Esta notificación permite la investigación detallada de los casos, facilitando la comprensión de la ocurrencia, causalidad y evolución de los eventos. Además, permite identificar los riesgos, los grupos poblacionales más vulnerables y las realidades contextuales en las que se insertan, considerando factores institucionales/organizacionales, ambientales, políticos y socioeconómicos^(6-11)^.

El período analizado en este estudio, de enero a diciembre de 2021, estuvo marcado por la mayor campaña de vacunación de la historia de Brasil, impulsada por el Ministerio de Salud. A lo largo de ese año se aplicaron más de 350 millones de dosis de la vacuna contra la COVID-19 en todo el país. Esta movilización masiva resultó en un aumento significativo en el volumen de notificaciones, lo que refleja la alta demanda de la vacuna y la intensificación de las acciones de vacunación^(12)^. Este contexto resalta la importancia de la notificación como un indicador de desempeño de la vigilancia, permitiendo la identificación de patrones y brechas en el sistema de registro y monitoreo de eventos adversos (errores de digitación, campos incompletos y casos de subnotificaciones)^(8-11,13-14)^.

Dada la importancia de la vigilancia de ESAVI, el Plan de Acción Mundial sobre Vacunas (GVAP) estableció la tasa de notificación como indicador de desempeño para evaluar el progreso de la vigilancia pasiva de la seguridad de las vacunas^(13-14)^. La tasa de notificación analiza el número de notificaciones de ESAVI en una determinada región, en función de su población, y el estándar es que la región debe alcanzar al menos 10 notificaciones^(15-16)^. Los resultados de esta medida son útiles para análisis comparativos entre países y regiones, y pueden apoyar la definición de metas para la mejora progresiva de la vigilancia de ESAVI, adaptadas a las particularidades de cada localidad^(16)^.

Un estudio comparativo de las tendencias de las tasas de notificación de ESAVI a nivel global, entre los años 2000 y 2015, observó oscilaciones positivas en las tasas, indicando que el número de países con tasas de notificación de ESAVI superiores a 10 aumentó de 8 (4%) en 2000 a 81 (42%) en 2015^(15)^. Siguiendo en la misma línea, una investigación identificó avances globales en la proporción de países que reportaron ESAVI entre 2010 y 2019, aumentando del 41,2% al 56,2%^(17)^. Además, un estudio realizado en Estados Unidos analizó la tasa de notificación con base en la población, revelando una tendencia creciente en las notificaciones de ESAVI en el país^(16)^.

En Brasil, no se identificaron estudios que abordaran la tasa de notificación para analizar el desempeño de los servicios de inmunización en la vigilancia pasiva de ESAVI. Cuando se trata de notificaciones ESAVI de vacunas COVID-19 en el país, estas se realizan por medio de *e-SUS Notifica*, *software* que utiliza una ficha de notificación/investigación, luego de buscar espontáneamente el identificador del evento^(18-19)^. El seguimiento de información sobre ESAVI permite identificar notificaciones, monitorear casos y mejorar la calidad de la información^(8-11)^.

Sin embargo, la subnotificación de ESAVI ha sido un desafío preocupante, lo que pone de relieve la necesidad de perfeccionar los sistemas de vigilancia en los municipios brasileños^(9,20)^. La subnotificación puede atribuirse a varios factores, como infraestructura de salud insuficiente, conducta y falta de capacitación adecuada de los profesionales, deficiencia de recursos humanos, falta de insumos, falta de supervisión de enfermería, sobrecarga de trabajo, falta de motivación, falta de concientización de la población, y aspectos geográficos, culturales y económicos de la región^(21-26)^.

Ante este escenario, una atención singular y una planificación estratégica coherente con las características de cada localidad brasileña se vuelven esenciales para avanzar en la vigilancia pasiva de la seguridad de las vacunas.

Este tipo de vigilancia busca comprender las interacciones entre los medicamentos, el medio ambiente y la salud humana, utilizando técnicas espaciales para monitorear e identificar áreas de riesgo potencial e implementar medidas preventivas y correctivas dirigidas a determinadas regiones. La geofarmacovigilancia de vacunas realiza un seguimiento espacial de ESAVI o de cualquier otro problema relacionado con la vacunación o inmunización, ayudando a detectar posibles tendencias, clústeres o zonas con mayor incidencia del evento^(27)^.

El presente estudio analizó la distribución espacial de la tasa de notificación de eventos supuestamente atribuibles a la vacunación o inmunización contra la COVID-19 y factores asociados al cumplimiento de la meta recomendada, según el Plan de Acción Mundial sobre Vacunas.

## Método

### Diseño del estudio

Este es un estudio ecológico, guiado por la herramienta *SQUIRE* 2.0 (*Standards for Quality Improvement Reporting Excellence*).

### Escenario del estudio

El estudio se realizó en Minas Gerais, el segundo estado más poblado de Brasil, con una población estimada de 21.411.923 millones de habitantes, un grado de urbanización del 85,29%, un Índice de Desarrollo Humano (IDH) medio-alto, y una economía diversificada^(28)^. La elección de Minas Gerais para realizar este estudio se justifica por su gran diversidad demográfica, social y económica, además de albergar el mayor número de municipios del país, lo que ofrece un escenario rico y complejo para el análisis. Asimismo, la alianza entre la *Secretaria de Estado de Saúde* (SES-MG) y la *Universidade Federal de São João del-Rei* (UFSJ), por medio de proyectos colaborativos, garantiza una mayor aplicabilidad e impacto de los resultados.

Los 853 municipios están distribuidos en 14 regiones de salud: Sur (3101), Centro Sur (3102), Centro (3103), Jequitinhonha (3104), Oeste (3105), Este (3106), Sudeste (3107), Norte (3108), Noroeste (3109), Este Sur (3110), Nordeste (3111), Triángulo del Sur (3112), Triángulo del Norte (3113) y Vale do Aço (3114) (Figura 1). Estas, a su vez, abarcan 89 microrregiones de salud y 28 Superintendencias/Gerencias Regionales de Salud^(28-29)^.

Para este estudio se establecieron como unidades territoriales de análisis los 853 municipios de las 14 macrorregiones del estado (Figura 1).

**Figura 1 -:** Regiones de salud del estado de Minas Gerais, Brasil, 2022

### Población y recolección de datos

Se analizaron notificaciones de ESAVI de vacunas contra la COVID-19, con y sin cierre, disponibles en la plataforma electrónica del Departamento de Informática del *Sistema Único de Saúde* (DATASUS), sistema *e-SUS Notifica* ^(19)^, entre enero y diciembre de 2021. La elección de la vacunación contra la COVID-19 fue motivada por la relevancia y urgencia impuestas por la pandemia, además de ser un evento reciente y de gran impacto en la salud pública. En esta investigación se incluyeron las 34.027 notificaciones de ESAVI y se excluyeron los errores de vacunación (n= 2663).

La variable desenlace fue el número de notificaciones de ESAVI, con y sin cierre. La notificación de ESAVI se utiliza para detectar riesgos relacionados con vacunas o prácticas de vacunación, empleando la ficha de notificación/investigación de eventos adversos^(4)^. En este estudio se calculó la tasa de notificación de ESAVI, que consiste en el número de notificaciones de ESAVI dividido por la población de una determinada región, multiplicado por 100.000. El estándar utilizado para evaluar la tasa de notificación fue el número mínimo de 10 notificaciones por 100.000 habitantes^(13-16)^.

Para identificar los factores asociados al cumplimiento de las tasas de notificación de ESAVI, se seleccionaron variables de exposición del *Cadastro Nacional dos Estabelecimentos de Saúde do Brasil* (CNES), de la base de datos del *Instituto Brasileiro de Geografia e Estatística* (IBGE), y de la *Fundação João Pinheiro* (FJP), que se incluyeron en los análisis de regresión logística (Figura 2).

**Figura 2 -:** Descripción de las variables de exposición consideradas para el estudio. Minas Gerais, Brasil, 2023

### Procesamiento y análisis de datos

Inicialmente, en la fase exploratoria, se utilizó el *software Microsoft Office Excel*, versión 2016, para verificar la consistencia y la calidad de los registros de las notificaciones de ESAVI.

Además, se utilizó la tasa de notificación de ESAVI (número de notificaciones de ESAVI dividido por la población de una determinada región, multiplicado por 100.000) para crear los mapas referentes a los análisis Gi* de Getis y Ord y de Regresión geográficamente ponderada (*Geographically weighted regression* - GWR)^(30-31)^.

La herramienta utilizada para determinar la distribución de las tasas de notificación de ESAVI en el espacio fue el *software GeoDa 1.20.* Se trata de un instrumento de *software* libre y de código diseñado para llevar a cabo métodos de análisis espacial exploratorio de datos (ESDA), como estadísticas de autocorrelación espacial. También ofrece un módulo estadístico para el análisis Gi* de Getis y Ord. Y el análisis GWR fue realizado por el *software* libre R 4.3, para computación estadística y gráficos^(32-34)^.

Para todos los análisis espaciales se utilizó la matriz de vecindad del tipo reina de orden 1. Se consideraron todos los municipios que comparten frontera directa y en todas las direcciones.

Se utilizó el análisis Gi* de Getis y Ord para identificar aglomerados de tasas de notificación de ESAVI resultantes de las vacunas anti-COVID-19. La estadística consiste en la razón entre el promedio ponderado de los valores de las localidades vecinas y la suma de todos los valores, incluido el valor de la localidad específica que se analiza en una ventana analítica. Por lo tanto, valores por encima del promedio se clasifican como áreas de alta ocurrencia, mientras que valores por debajo del promedio sugieren baja ocurrencia. Este análisis resulta en dos clasificaciones: una para los municipios con valores altos, estadísticamente significativos (área de alta ocurrencia/área caliente - *Hotspot*) y otra para los municipios con valores bajos, también estadísticamente significativos (área de baja ocurrencia/área fría - *Coldspot*)^(31,35)^.

Para identificar los factores asociados con la tasa de notificación de ESAVI, se elaboró una regresión geográficamente ponderada, considerando variables explicativas que caracterizaran los municipios analizados (Figura 2). Este enfoque de modelización implementa, dentro de una estructura de regresión lineal local (R²), el efecto de decaimiento de distancia, siguiendo el principio de la primera ley de la geografía, es decir, los coeficientes de la regresión no son estacionarios, sino que se estiman individualmente para cada unidad de análisis espacial (municipios), y se aplica un efecto suavizador, considerando una matriz de vecinos^(31)^.

Para elaborar los mapas coropléticos, se utilizó la base cartográfica del estado de Minas Gerais, incluidos sus respectivos municipios y macrorregiones, obtenida de forma gratuita en el sitio *web* del IBGE y procesada mediante el *software* ArcGIS 10.8.

El presente estudio utilizó datos de dominio público y de acceso irrestricto, sin ninguna identificación de los individuos participantes en la investigación. Por ende, no fue necesaria su presentación para la evaluación del Comité de Ética en Investigación (CEI).

## Resultados

De los 853 municipios del estado de Minas Gerais, el 20,3% (n= 173) no alcanzaron la meta recomendada de un mínimo de 10 notificaciones de ESAVI (tasa de notificación). Los municipios de las macrorregiones Noroeste, Jequitinhonha y Vale do Aço presentaron las tasas de notificación más altas del estado. Por otro lado, los municipios de las macrorregiones Este, Este Sur y Oeste tuvieron las tasas más bajas (Figura 3). Se observa una gran heterogeneidad en el cumplimiento de la meta de vigilancia de ESAVI entre los municipios de Minas Gerais.

**Figura 3 -:** Cumplimiento de la meta de las tasas de notificación de eventos supuestamente atribuibles a la vacunación o inmunización contra la COVID-19. Minas Gerais, Brasil, 2021

En el análisis Gi* de Getis y Ord, se identificó una distribución heterogénea de las tasas de notificación de ESAVI resultantes de las vacunas contra la COVID-19 en todo el estado de Minas Gerais. Se destaca la presencia de dos grandes aglomerados con baja tasa de notificación, identificados como áreas frías, en la macrorregión Norte. Los aglomerados con alta tasa de notificación, considerados áreas calientes, incluyen las macrorregiones Noroeste, Centro y Triángulo del Norte (Figura 4).

**Figura 4 -:** Aglomerados espaciales de las tasas de notificación de eventos supuestamente atribuibles a la vacunación o inmunización contra la COVID-19. Minas Gerais, Brasil, 2021

La regresión lineal, considerando la tasa de notificación de ESAVI y el número de profesionales de enfermería por habitante (coeficiente de regresión= 0,644), sugiere que existe una asociación positiva entre las variables, es decir, a medida que aumenta el número de profesionales de enfermería, la tasa de notificación de ESAVI también tiende a aumentar, y el valor de p fue < 0,01. Otra variable destacada fue el porcentaje de familias residentes en áreas rurales, que presentó un coeficiente negativo (-0,013), lo que indica que, a medida que aumenta el número de familias residentes en áreas rurales, disminuye la tasa de notificación de ESAVI, y el valor de p fue < 0,01.

El valor del Criterio de Información de Akaike (AIC) fue calculado como 3617.779, lo que proporciona una medida de la calidad del modelo, considerando el equilibrio entre el ajuste y la complejidad. Cuanto menor sea el valor del AIC, mejor será el ajuste del modelo. Además, el coeficiente de determinación (R²) se calculó como 0,03, lo que indica que aproximadamente el 3% de la variación en la tasa de notificación de ESAVI puede explicarse por las variables incluidas en el modelo (*Akaike Information Criterion*: 3617.779 y R^2^: 0.03).

La asociación de la tasa de notificación de ESAVI en relación con las variables número de profesionales de enfermería por habitante y porcentaje de familias residentes en áreas rurales está distribuida de forma heterogénea en todo el estado de Minas Gerais, es decir, cada variable tiene diferentes asociaciones (valor del coeficiente) según las regiones (Figura 5).

Al analizar la regresión geográficamente ponderada, se observó que la tasa de notificación de ESAVI tiene una asociación positiva, considerando el número de profesionales de enfermería por habitante (variable A), en las regiones Norte y Sur del estado.

En relación con el coeficiente de regresión del porcentaje de familias residentes en áreas rurales (variable B), se identificó una asociación negativa en gran parte del estado, siendo el valor más bajo localizado en una vasta área en la región Norte, seguida por la región Oeste.

El R² local (variable C) demuestra la capacidad de explicación de la ocurrencia de la tasa de notificación de ESAVI derivada de las vacunas contra la COVID-19 en Minas Gerais. La capacidad de explicación de la asociación entre las variables explicativas y la tasa de notificación de ESAVI es mayor en las regiones Norte y Sur del estado y menor en la región Centro.

**Figura 5 -:** Análisis de regresión espacial geográficamente ponderada (GWR) para la tasa de notificación de eventos supuestamente atribuibles a la vacunación o inmunización contra la COVID-19 en los municipios de Minas Gerais. Minas Gerais, Brasil, 2021

*A = Número de profesionales de enfermería por habitante; ^†^B = Porcentaje de familias residentes en áreas rurales; ^‡^C = R^2^ local

## Discusión

En Minas Gerais, se observaron aglomerados con bajas tasas de notificación, o incluso sin ninguna notificación, con respecto a la vigilancia de los ESAVI derivados de las vacunas contra la COVID-19. Además, se constató que, a pesar de que todas las macrorregiones cumplieron con la meta de la tasa de notificación, el 20,3% de sus municipios no alcanzaron este objetivo. Dos factores estuvieron asociados con las tasas de notificación: el número de profesionales de enfermería por habitante y el porcentaje de familias residentes en áreas rurales. La distribución de estas variables asociadas a la tasa de notificación de ESAVI se presentó de manera heterogénea en todo el estado.

Estudios indican que la heterogeneidad en las tasas de notificación de ESAVI está relacionada con la ubicación geográfica, el nivel de concientización y capacitación de los profesionales de la salud, así como el conocimiento de la población sobre la notificación de eventos adversos^(15,36-37)^. Estos factores refuerzan la importancia de estrategias educativas y de concientización de la población, combinadas con la capacitación de profesionales de la salud, para promover un sistema robusto de vigilancia de eventos adversos y, en consecuencia, seguridad y confianza en las prácticas de vacunación. Otros factores asociados con ESAVI incluyen el aumento de notificaciones de eventos adversos en los primeros años de licencia de nuevas vacunas y el aumento de notificaciones luego de la divulgación de un evento adverso específico^(15)^.

En cuanto a la ubicación geográfica, Minas Gerais tiene una vasta extensión territorial y regiones con diferentes desarrollos socioeconómicos^(38)^. Las regiones Noroeste y Vale do Aço, que tienen los mejores indicadores socioeconómicos, tuvieron las tasas más altas de notificación de ESAVI de COVID-19. La región de Jequitinhonha, a pesar de destacarse por sus bajos indicadores socioeconómicos, también presentó altas tasas de notificación de ESAVI. Se identificaron aglomeraciones con bajas tasas de notificación en la región Norte del estado. El Norte de Minas, en comparación con otras regiones del estado, ocupa una posición desventajosa debido a sus bajos indicadores socioeconómicos, como el ingreso promedio per cápita y la vulnerabilidad social^(38-39)^.

Una investigación internacional reforzó que las regiones más desarrolladas tienden a reportar más ESAVI que las menos desarrolladas^(17)^. Se supone que las regiones más desarrolladas cuentan con una infraestructura sanitaria más adecuada, lo que puede facilitar el acceso a la vacunación, aumentar la cobertura de vacunación y, en consecuencia, aumentar la notificación de eventos adversos. Además, estas regiones tienden a contar con estrategias de comunicación más efectivas, capaces de esclarecer y alentar a la población a informar sobre la ocurrencia de eventos posvacunación. Por otro lado, a pesar de las disparidades socioeconómicas en la región de Jequitinhonha, es posible suponer que la región ha mostrado una mayor conciencia y compromiso de la población con respecto a la notificación de eventos adversos de la COVID-19, o incluso esfuerzos más intensos para la sensibilización y el seguimiento posvacunación.

En el territorio brasileño también se observa heterogeneidad en las tasas de notificación. Solamente el 46% de los municipios brasileños realizaron al menos una notificación de ESAVI, y la mayoría de los municipios silenciosos se concentraron en las regiones Norte (23,6%) y Nordeste (38,6%) del país^(9)^. Asimismo, las incidencias más bajas de eventos adversos reportados se observaron en la región Norte del país^(9,40)^.

Este evento está presente más allá de las fronteras brasileñas. Un estudio realizado con 194 países demostró que existe gran heterogeneidad en la notificación de ESAVI entre regiones y países^(15)^. Los resultados de este estudio mostraron que solamente el 64% de los países reportaron ESAVI a través del Formulario de Notificación Conjunta de Inmunización de la Organización Mundial de la Salud (OMS). Entre los países de la región de las Américas y África, el 83% y el 51% hicieron al menos una notificación, respectivamente^(15)^. Los autores también señalaron la existencia de países silenciosos. Los resultados de nuestra investigación también identificaron algunos municipios de Minas Gerais que comparten este silencio respecto de las notificaciones de ESAVI.

Las disparidades regionales, tanto geográficas como económicas, refuerzan la importancia de los análisis espaciales para identificar áreas silenciosas de notificación y crear intervenciones dirigidas a la región, ya que estas diferencias pueden comprometer las acciones y servicios proporcionados por los municipios^(38-39,41-44)^. Es importante considerar que cada localidad se encuentra en diferentes etapas de desarrollo, por lo que el propósito principal de la tasa de notificación de ESAVI es establecer un estándar para evaluar el progreso del desempeño de los sistemas de vigilancia de ESAVI^(13-14)^.

Independientemente de los factores que contribuyen a las diferencias en los requisitos de notificación de ESAVI entre países y regiones, la vigilancia de ESAVI se caracteriza por un alto grado de subnotificación^(43)^. Esta falta de datos constituye un obstáculo significativo para formular estrategias efectivas en regiones silenciosas^(44)^.

En la vigilancia pasiva, la falta de notificaciones de ESAVI, por ejemplo, no implica necesariamente la ausencia de estos eventos. La subnotificación, entendida como la falta de registro o notificación oficial de los casos efectivamente ocurridos, resulta en una subestimación de las cifras reales de incidencia o prevalencia de la enfermedad y/o evento^(45)^. Es evidente que la subnotificación no está vinculada únicamente a las diferencias regionales (aspectos geográficos, culturales y económicos); también puede atribuirse a otros factores, como la falta de educación continua de los profesionales, la insuficiencia de recursos humanos, la ausencia de supervisión de enfermería, la sobrecarga de trabajo, la desmotivación y la falta de triaje vacunal^(7,9,46-47)^. Estos factores se atribuyen a la capacidad y formación profesional para actuar en los servicios de inmunización.

En el contexto brasileño, es el equipo de enfermería el principal responsable de las actividades en la sala de vacunación. La Regresión geográficamente ponderada (GWR) mostró asociación positiva entre la tasa de notificación de ESAVI y el número de profesionales de enfermería por habitante. La literatura destaca que las acciones adoptadas por estos profesionales juegan un papel crucial en la prevención de la subnotificación de ESAVI^(20-21,24)^. La realización de triaje vacunal, con foco en brindar orientación sobre las vacunas administradas y los posibles ESAVI causados por las mismas, es un procedimiento que incrementa las notificaciones, la confianza en los inmunobiológicos y el avance de la vigilancia pasiva^(20-21,24,48)^.

Estudios internacionales también destacaron que la conducta de los profesionales de la salud que realizan triaje vacunal aumenta la notificación de eventos adversos y, en consecuencia, su incidencia^(37,48-50)^. Sin embargo, un estudio realizado en Mysuru, India, encontró que más de la mitad (73,37%) de los profesionales que asistieron a un ESAVI no lo notificaron^(50)^. Hay una presencia de subnotificación del evento, asociada a dudas de los profesionales sobre los eventos que deben ser reportados, con foco en eventos graves y menos frecuentes^(11)^.

Además de las dudas relacionadas con los ESAVI, los profesionales tienen temores de notificar debido al miedo a represalias, a la falta de información sobre la persona vacunada, a la sobrecarga de trabajo y a los errores de inmunización^(22-24,51)^. Esto refuerza la necesidad de capacitar a los profesionales en la vigilancia de los ESAVI, incluyendo el uso de triaje vacunal para la sensibilización de la comunidad, la educación continua y la supervisión^(29-30)^.

Por último, la subnotificación de ESAVI también puede ocurrir debido a cuestiones tecnológicas, como fallos en los *softwares*, problemas en la infraestructura tecnológica, conectividad limitada, falta de interoperabilidad, sobrecarga y estandarización de los sistemas de información en salud (SIS), lo que puede dificultar la recopilación y transmisión de datos^(9,52-54)^. Estas cuestiones tecnológicas experimentadas en los servicios pueden intensificar la subnotificación de los ESAVI y afectar la calidad de la información. Debido a la pandemia, por ejemplo, gran parte de los sistemas tuvo que manejar un volumen elevado de datos, lo que influyó directamente en la incompletitud de los datos y en la inclusión de errores, lo que podría resultar en subnotificaciones^(54)^.

Otro factor asociado a la tasa de notificación de ESAVI fue el porcentaje de familias residentes en áreas rurales. La literatura demuestra que los habitantes de estas localidades enfrentan desafíos considerables para acceder a los centros de vacunación. La distancia entre las viviendas y los servicios de salud, la dificultad de acceso al transporte público, la falta de mantenimiento en las carreteras y la necesidad de recorrer largas distancias hasta la unidad de salud son barreras geográficas que impactan directamente el proceso de vacunación^(7,25,56-57)^. Por consiguiente, estos factores dificultan el monitoreo de los casos de ESAVI, lo que resulta en una reducción de las notificaciones de estos eventos.

Este estudio se basa en datos secundarios, lo que implica algunas limitaciones que deben ser consideradas. La calidad de los resultados está directamente relacionada con la exactitud y confiabilidad de los datos originales, y puede verse afectada por posibles inconsistencias o subnotificaciones. Además, la incorporación de las notificaciones en el *e-SUS Notifica* es realizada por diversos profesionales, lo que puede generar variaciones en los registros. Por lo tanto, al interpretar los resultados de este estudio, es crucial considerar estas limitaciones y evaluar cómo pueden influir en las instrucciones y la aplicabilidad de los resultados en diferentes contextos y escenarios.

Sin embargo, los hallazgos identificados demuestran el potencial de la técnica de análisis espacial, ya que permitió identificar las macrorregiones de salud de Minas Gerais que requieren intervenciones prioritarias para avanzar en la vigilancia de los ESAVI. Y la fortaleza del estudio radica en el avance del conocimiento científico al proporcionar un análisis detallado de la distribución espacial de las notificaciones de ESAVI relacionadas con la COVID-19 y los factores asociados. Mediante la aplicación de métodos avanzados de análisis espacial, como el análisis Gi* de Getis y Ord y la Regresión geográficamente ponderada, el estudio identificó patrones geográficos relevantes en las tasas de notificación y revela disparidades regionales, destacando áreas con baja adherencia a la notificación de eventos. Se destaca la importancia de factores como el número de profesionales de enfermería por habitante y el porcentaje de familias en áreas rurales en la influencia de estas tasas, lo que contribuye a una comprensión más profunda de los determinantes sociales y estructurales que afectan la notificación de eventos.

## Conclusión

El análisis de la distribución espacial de las notificaciones de ESAVI en el estado identificó municipios silenciosos que no alcanzaron el valor mínimo de notificaciones de ESAVI. La presencia de aglomerados señala la necesidad de implementar estrategias integradas, adaptadas a las particularidades de cada región, para mejorar la vigilancia de la seguridad de las vacunas contra la COVID-19 en todo el estado brasileño.

Los resultados de este artículo son útiles para la optimización de estrategias de vigilancia. Las Superintendencias/Gerencias Regionales de Salud pueden utilizar los datos proporcionados para identificar las áreas con mayores y menores tasas de notificación de ESAVI, dirigir recursos adicionales, monitorear de cerca las prácticas de vacunación e implementar campañas educativas específicas en esas regiones.

Además, el análisis de la heterogeneidad espacial reveló patrones socioeconómicos asociados a estas ocurrencias, permitiendo estrategias más enfocadas hacia grupos vulnerables. La colaboración interinstitucional también se hace posible, facilitando el intercambio de mejores prácticas entre regiones. En resumen, los datos proporcionaron una base sólida para la toma de decisiones informadas, fortaleciendo la capacidad de las superintendencias de salud para promover la seguridad y eficacia en el monitoreo de las vacunas.
